# Mitochondrial genomes of two phlebotomine sand flies, *Phlebotomus chinensis* and *Phlebotomus papatasi* (Diptera: Nematocera), the first representatives from the family Psychodidae

**DOI:** 10.1186/s13071-015-1081-1

**Published:** 2015-09-17

**Authors:** Fei Ye, Ting Liu, Stanley D. King, Ping You

**Affiliations:** Co-Innovation Center for Qinba regions’ sustainable development, College of Life Science, Shaanxi Normal University, Xi’an, 710062 China; Department of Biology, Dalhousie University, Halifax, NS Canada B3H 4J1

**Keywords:** *Phlebotomus chinensis*, *Phlebotomus papatasi*, Leishmaniasis, Mitochondrial genome, Psychodidae, Phylogenetic analysis

## Abstract

**Background:**

Leishmaniasis is a worldwide but neglected disease of humans and animal transmitted by sand flies, vectors that also transmit other important diseases. Mitochondrial genomes contain abundant information for population genetic and phylogenetic studies, important in disease management. However, the available mitochondrial sequences of these crucial vectors are limited, emphasizing the need for developing more mitochondrial genetic markers.

**Methods:**

The complete mitochondrial genome of *Phlebotomus chinensis* was amplified in eight fragments and sequenced using primer walking. The mitochondrial genome of *Phlebotomus papatasi* was reconstructed from whole-genome sequencing data available on Genbank. The phylogenetic relationship of 24 selected representatives of Diptera was deduced from codon positions 1 and 2 for 13 protein coding genes, using Bayesian inference (BI) and maximum likelihood (ML) methods.

**Results:**

We provide the first *Phlebotomus* (*P. chinensis* and *P. papatasi*) mitochondrial genomes. Both genomes contain 13 protein-coding genes, 22 transfer RNA genes, two ribosomal RNA genes, and an A + T-rich region. The gene order of *Phlebotomus* mitochondrial genomes is identical with the ancestral gene order of insect. Phylogenetic analyses demonstrated that Psychodidae and Tanyderidae are sister taxa. Potential markers for population genetic study of *Phlebotomus* species were also revealed.

**Conclusion:**

The generated mitochondrial genomes of *P. chinensis* and *P. papatasi* represent a useful resource for comparative genomic studies and provide valuable future markers for the population genetic study of these important *Leishmania* vectors. Our results also preliminary demonstrate the phylogenetic placement of Psychodidae based on their mitochondrial genomes.

## Background

Phlebotomine sand flies are small insects in the family Psychodidae, and are important vectors of human disease including protozoal parasite, bacteria, and viruses [1] making these insects a global public health concern. Leishmaniasis is one of the world’s most neglected diseases transmitted by phlebotomine sand flies, causing significant mortality and morbidity in more than 80 countries of both the Old and New World. The majority of Old World vector species belong to the genus *Phlebotomus* (42 vector species) while the New World is dominated by the genus *Lutzomyia* (56 vector species) [2]. Of *Phlebotomus* species, two are of particular interest; *Phlebotomus chinensis* and *Phlebotomus papatasi. Phlebotomus chinensis*, the main vector of mountainous sub-type of zoonotic visceral leishmaniasis, has wide geographical distribution extending from the Yangtze River to northeast China [3–5]. In recent years, the number of visceral leishmaniasis (VL) cases and its endemic foci has increased (54.37 % and 41.86 % respectively) compared to that of the 1990s in China. Until now, six provinces/autonomous regions still reported autochthonous cases. The area of mountainous sub-type of zoonotic VL covers four provinces which possess almost half of the total cases [6–8]. Prevention and control of vector *P. chinensis* is important to reduce the public health threat of VL in endemic regions. *Phlebotomus papatasi* is the vector of sand fly fever and zoonotic cutaneous leishmaniasis in Middle East and Mediterranean regions and is also an important model organism used to study sand flies-host-parasite interactions [9–12].

In recent years, the mitochondrial genome has become increasingly important in phylogenetic analysis, biological identification and population studies, due to its rapid evolutionary rate, low recombination and maternal inheritance [13, 14]. Although microsatellites and individual gene sequences, such as *Cytb* and *ND4*, have been used for sand fly studies in the past [15–17], the mitochondrial genome of phlebotomine sand flies has gone largely unstudied which is surprising given their pathogenic potential. The complete mitochondrial genome contains important information not available in examining individual genes, including genome-level characteristics for phylogenetic reconstruction. Additionally, due to the varying rates of gene evolution, the mitochondrial genome can also provide various molecular markers for studying phylogenetic relationships at different taxonomic levels, including intraspecies population structure.

Despite these benefits, information on the mitochondrial genomes of Diptera is still limited, especially for representatives of Nematocera. Most of these genomes are sequenced by long PCR with primer walking method. As the widespread application of next-generation sequencing (NGS), long PCR with next-generation sequencing, and direct shotgun sequencing methods has been utilized in mitochondrial genomes determination [18, 19]. Although the Sanger sequencing is still the indispensable method, the NGS method is relatively fast and inexpensive especially for direct shotgun sequencing method. In fact, this method for reconstruction of mitochondrial genomes becomes one of the simplest approaches. In the present study, we determined the complete mitochondrial genome of two important *Leishmania* vectors, *P. chinensis* and *P. papatasi* with long PCR with primer walking method and reconstruction from direct shotgun sequencing data respectively, reporting their genome features and analyzing the overall phylogenetic status of Psychodidae within Diptera. The addition of new mitochondrial genomes from nematoceran species would be of critical importance in understanding the evolution of Nematocera mitochondrial genome and examining the phylogeny in the Nematocera and Diptera.

## Methods

### Specimen collection and DNA extraction

Specimens of *P. chinensis* were collected from Wen County (104.25°E, 33.18°N), Gansu province, China. All specimens were preserved in 95 % ethanol and stored at −20 °C until DNA extraction. DNA was extracted from the single adult *P. chinensis* using the TIANamp Micro DNA Kit (Tiangen Biotech, Beijing, China) according to the manufacturer’s protocol.

### Mitochondrial genome determination

The complete mitochondrial genome of *P. chinensis* was amplified in eight overlapping PCR fragments from a single adult. First, six fragments were amplified using previously published primers (Table 1). Then, from the generated sequences, two specific primers were designed for amplifying overlapping fragments spanning the whole mitochondrial genome. Short fragments (<2 kb) were amplified using TaKaRa rTaq (not proof-reading; Takara Biotech, Dalian, China; http://www.takara.com.cn) with the following cycling conditions: an initial denaturation for 1 min at 93 °C, followed by 35 cycles of 10 s at 92 °C, 1.5 min at 48–57 °C, 1–2 min at 72 °C, and final extension of 6 min at 72 °C. Long fragments (>2 kb) were amplified using TaKaRa LA Taq (proof-reading; Takara Biotech, Dalian, China; http://www.takara.com.cn) under the following cycling conditions: an initial denaturation for 1 min at 94 °C, followed by 40 cycles of 20 s at 93 °C, 30 s at 48–54 °C, 3–6 min at 68 °C, and final extension of 10 min at 68 °C. After purification with PCR Purification Kit (Sangon Biotech, Shanghai, China), all PCR products were sequenced directly with the PCR primers and internal primers generated by primer walking. The complete mitochondrial genome of *P. papatasi* was reconstructed from 454 sequencing data publicly available in the Sequence Read Archive (SRA) of GenBank (Accession number: SRX027115). Reconstruction was done by the baiting and iterative mapping approach of [20] using software MITObim v1.7 with default parameters [21, 22]. The mitochondrial genome of *P. chinensis* as the reference sequence.

**Table 1:** List of PCR primer combinations used to amplify the mitochondrial genome of *Phlebotomus chinensis*

Primer name	Gene	Sequence(5′–3′)	Reference
1F^a^(SR-J14610)	*rrnS*	ATAATAGGGTATCTAATCCTAGT	[62]
1R^a^(HCO2198)	*COI*	TAAACTTCAGGGTGACCAAAAAATCA	[63]
2F^a^(LCO1490)	*COI*	GGTCAACAAATCATAAAGATATTGG	[63]
2R^a^(C2-N3665)	*COII*	CCACAAATTTCTGAACATTG	[62]
3F	*COII*	TTAGATGTCGATAACCGAAT	This study
3R	*COIII*	AATGTAGTCCTTGAAATGTG	This study
4F(C3-J4792)	*COIII*	GTTGATTATAGACCWTGRCC	[62]
4R(TF-N6384)	*trnF*	TATATTTAGAGYATRAYAYTGAAG	[62]
5F^a^(TN-J6155)	*trnN*	TTTAATTGAARCCAAAAAGAGG	[62]
5R^a^(N4L-N9629)	*ND4L*	GTTTGTGAGGGWGYTTTRGG	[62]
6F(N4-J9172)	*ND4*	CGCTCAGGYTGRTACCCYCA	[62]
6R(CB-N11010)	*Cytb*	TATCTACAGCRAATCCYCCYCA	[62]
7F^a^	*Cytb*	CTTGATCTATTGGAACATT	This study
7R^a^	*rrnL*	TACCTTAGGGATAACAGCG	This study
8F^a^(LR-J12888)	*rrnL*	CCGGTCTGAACTCARATCATGTA	[62]
8R^a^(SR-N14745)	*rrnS*	GTGCCAGCAGYYGCGGTTANAC	[62]

^a^The PCR primers for the long PCR fragment (>2 kb)

### Sequence analyses

Contiguous sequence fragments were assembled using Staden Package v1.7.0 [23]. Protein coding genes (PCGs) and ribosomal RNA (rRNA) genes were identified based on homologous regions of other dipteran insects using the Clustal X [24]. Transfer RNAs (tRNA) and their potential cloverleaf structures were identified by tRNAscan-SE 1.21 [25]. The secondary structure of the two rRNA genes was determined mainly by comparison with the published rRNA secondary structures of *Drosophila melanogaster* and *Drosophila virilis* [26]. Tandem Repeat Finder v4.07 was used to identify tandem repeats in A + T-rich region [27]. The base composition and codon usage were calculated with MEGA 5.1 [28]. AT and GC skew were calculated according to the formulae: AT skew = (fA − fT) / (fA + fT) and GC skew = (fG − fC) / (fG + fC). Sliding window analyses were performed using DnaSP v5 [29]. A sliding window of 500 bp (in 25 bp overlapping steps) was used to estimate nucleotide diversity Pi (π) across the alignment of *P. chinensis*, *P. papatasi* and *Lutzomyia umbratilis* [30] mitochondrial genomes excluding the A + T-rich region.

### Phylogenetic analyses

For the phylogenetic analyses, a total of 24 representative species from Diptera were used to build the alignment (Table 2), with *Bittacus pilicornis* used as the outgroup (Mecoptera). All 13 PCGs were extracted and translated (excluding the stop codon) using the invertebrate mitochondrial genetic code. We used the Clustal X for alignment of the inferred amino acid sequences. Then the alignments were transferred to the DNA sequences, and third codon positions removed. The best-fit model (GTR + Γ + I) was estimated by the Akaike information criterion in jModelTest [31]. MrBayes ver.3.1.2 [32] and RAxML ver.7.2.8 [33] were used to construct a maximum likelihood (ML) and bayesian inference (BI) phylogeny. For ML analyses, bootstrap analysis was performed with 1,000 replicates. For BI analyses, two sets of four chains were allowed to run simultaneously for 1,000,000 generations. Each set was sampled every 100 generations with a burn-in of 25 %. Stationarity was considered to be reached when the average standard deviation of split frequencies was less than 0.01.

**Table 2:** The species and their GenBank accession numbers used in our phylogenetic analyses

Species	Family	Accession number	Reference
*Phlebotomus chinensis*	Psychodidae	KR349297	This study
*Phlebotomus papatasi*	Psychodidae	KR349298	This study
*Lutzomyia umbratilis*	Psychodidae	KP702938	[30]
*Tipula abdominalis*	Tipulidae	JN861743	[34]
*Paracladura trichoptera*	Trichoceridae	JN861751	[34]
*Trichocera bimacula*	Trichoceridae	JN861750	[34]
*Ptychoptera sp.*	Ptychopteridae	JN861744	[34]
*Bittacomorphella fenderiana*	Ptychopteridae	JN861745	[34]
*Protoplasa fitchii*	Tanyderidae	JN861746	[34]
*Chironomus tepperi*	Chironomidae	JN861749	[34]
*Culicoides arakawae*	Ceratopogonidae	AB361004	[64]
*Culex pipiens*	Culicidae	NC_015079	Atyame et al. unpublished data
*Anopheles gambiae*	Culicidae	NC_002084	[65]
*Aedes albopictus*	Culicidae	AY072044	Ho et al. unpublished data
*Arachnocampa flava*	Keroplatidae	JN861748	[34]
*Cramptonomyia spenceri*	Pachyneuridae	JN861747	[34]
*Sylvicola fenestralis*	Anisopodidae	JN861752	[34]
*Cydistomyia duplonotata*	Tabanidae	DQ866052	[39]
*Simosyrphus grandicornis*	Syrphidae	DQ866050	[39]
*Ceratitis capitata*	Tephritidae	NC_000857	[66]
*Drosophila yakuba*	Drosophilidae	NC_001322	[67]
*Dermatobia hominis*	Oestridae	AY463155	Azeredo-Espin et al. unpublished data
*Cochliomyia hominivorax*	Calliphoridae	AF260826	[68]
*Haematobia irritans*	Muscidae	DQ029097	Lessinger et al. unpublished data
*Bittacus pilicornis*	Bittacidae	NC_015118	[69]

## Results and discussion

### Genome organization and composition

**Fig. 1:**
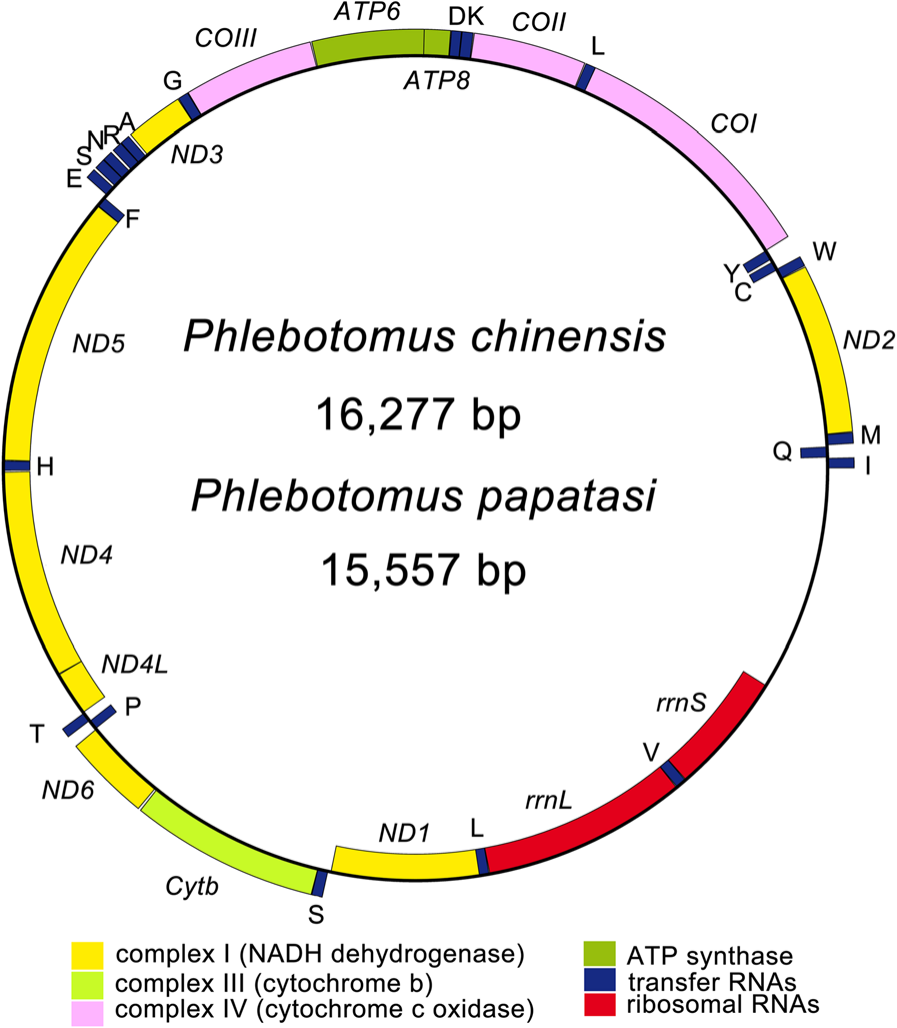
The gene map of the mitochondrial genomes of *Phlebotomus chinensis* and *Phlebotomus papatasi*

The circular mitochondrial genome of *P. chinensis* (GenBank accession number KR349297) is 16,277 bp in size. The complete mitochondrial genome of *P. papatasi* (GenBank accession number KR349298), 15,557 bp, was assembled from a total of 5579 reads identified as being of mitochondrial origin. An average per base estimated coverage of reconstructed mitochondrial genome of *P. papatasi* is ~ 209× based on the mean read length. The mitochondrial genome size differential stems mainly from the varying length of the A + T-rich region caused by variability in the number of tandem repeats. Consistent with published dipteran mitochondrial genomes, both *Phlebotomus* mitochondrial genomes contain 13 protein-coding genes (PCGs), 22 transfer RNA (tRNA) genes, two ribosomal RNA (rRNA) genes, and an A + T-rich region (Table 3). The majority-coding strand (J-strand) and the minority-coding strand (N-strand) encode 23 and 14 genes, respectively (Fig. 1). All the 37 genes share the identical arrangement with the hypothesized ancestral pancrustacean mitochondrial genome. The base composition of the *Phlebotomus* mitochondrial genome is biased toward A + T, with a total A + T content (J-strand) of 79.2 % and 77.5 % for *P. chinensis* and *P. papatasi*, respectively. We calculated the AT content, AT- and GC-skew of PCGs, RNAs and the control region of three sand flies (Table 4), and found that these regions also possess high A + T content, in particular the third codon position of PCG and control region is distinctly higher than that of other regions.

**Table 3:** The organization of the mitochondrial genome of *Phlebotomus chinensis* and *Phlebotomus papatasi*

Gene (region)	Strand	Position		Codon				Anticodon
		Pc	Pp	Pc		Pp		
				Start	Stop	Start	Stop	
*trnI*	J	1–65	1–65					GAT
*trnQ*	N	69–137	66–134					TTG
*trnM*	J	147–214	138–205					CAT
*ND2*	J	215–1237	206–1234	ATA	TAA	ATT	TAA	
*trnW*	J	1240–1304	1237–1302					TCA
*trnC*	N	1297–1358	1295–1357					GCA
*trnY*	N	1374–1440	1361–1428					GTA
*COI*	J	1439–2977	1427–2965	TCG	TAA	TCG	TAA	
*trnL* ^UUR^	J	2973–3037	2961–3025					TAA
*COII*	J	3040–3723	3028–3711	ATG	TAA	ATG	TAA	
*trnK*	J	3725–3795	3716–3786					CTT
*trnD*	J	3795–3858	3794–3858					GTC
*ATP8*	J	3859–4020	3859–4020	ATT	TAA	ATT	TAA	
*ATP6*	J	4014–4691	4014–4691	ATG	TAA	ATG	TAA	
*COIII*	J	4695–5483	4691–5479	ATG	TAA	ATG	TAA	
*trnG*	J	5483–5549	5483–5548					TCC
*ND3*	J	5550–5903	5549–5902	ATC	TAA	ATT	TAA	
*trnA*	J	5915–5976	5905–5967					TGC
*trnR*	J	5979–6041	5968–6031					TCG
*trnN*	J	6054–6118	6067–6130					GTT
*trnS* ^AGN^	J	6118–6186	6134–6202					GCT
*trnE*	J	6198–6263	6202–6266					TTC
*trnF*	N	6284–6350	6287–6351					GAA
*ND5*	N	6350–8089	6358–8097	ATA	TAA	ATA	TAG	
*trnH*	N	8090–8152	8098–8161					GTG
*ND4*	N	8159–9493	8162–9494	ATG	TAA	ATG	T	
*ND4L*	N	9493–9780	9494–9781	ATG	TAA	ATG	TAA	
*trnT*	J	9783–9844	9784–9847					TGT
*trnP*	N	9845–9908	9848–9911					TGG
*ND6*	J	9911–10438	9914–10438	ATA	TAA	ATA	TAA	
*Cytb*	J	10449–11588	10443–11582	ATG	TAG	ATG	TAG	
*trnS* ^UCN^	J	11587–11654	11581–11647					TGA
*ND1*	N	11670–12608	11663–12601	ATT	TAA	ATT	TAA	
*trnL* ^CUN^	N	12609–12674	12602–12667					TAG
*rrnL*	N	12675–13987	12668–13976					
*trnV*	N	13988–14058	13977–14047					TAC
*rrnS*	N	14059–14845	14048–14834					
A + T-rich region	—	14846–16277	14835–15557					

Notes: J and N refer to the majority and minority strand, respectively

Pc *Phlebotomus chinensis*, Pp *Phlebotomus papatasi*

**Table 4:** Composition and skewness of mitochondrial genomes of *Phlebotomus chinensis*, *Phlebotomus papatasi* and *Lutzomyia umbratilis*

Region	AT %			AT-skew			GC-skew		
	Pc	Pp	Lu	Pc	Pp	Lu	Pc	Pp	Lu
Whole genome	79.2	77.5	78.6	−0.014	−0.012	0.003	−0.248	−0.239	−0.209
Protein-coding genes	76.4	75.1	76.5	−0.167	−0.150	−0.125	−0.006	−0.016	0.036
First codon position	70.0	68.6	70.4	−0.088	−0.066	−0.060	0.225	0.209	0.265
Second codon position	67.8	67.5	67.6	−0.386	−0.389	−0.377	−0.159	−0.159	−0.143
Third codon position	91.3	89.0	91.4	−0.072	−0.039	0.008	−0.237	−0.241	−0.081
tRNA genes	80.8	79.8	79.4	−0.003	0.043	0.030	0.161	0.116	0.079
rRNA genes	84.3	83.4	84.1	−0.005	−0.012	−0.015	0.364	0.354	0.385
A + T-rich region	91.1	92.3	90.4	−0.074	−0.061	0.011	−0.938	−0.571	−0.576

Note: Pc *Phlebotomus chinensis*, Pp *Phlebotomus papatasi*, and Lu *Lutzomyia umbratilis*

### Protein-coding genes and codon usage

All the protein-coding genes of *P. chinensis* start with the typical ATN codon except for *COI* (Table 3). In comparison with *P. chinensis*, only *ND2* and *ND3* have the different start codon in *P. papatasi*. The start codon of *COI* in *P. chinensis* and *P. papatasi* is uncommon start codon TCG, which is also reported for *COI* in some nematoceran mitochondrial gneomes [34–36]. The conventional stop codons TAA or TAG were used in all the PCGs of *P. chinensis*, while *ND4* of *P. papatasi* terminates with the incomplete stop codon T. The conserved 7-bp overlap (ATGATAR) between *ATP8* and *ATP6* present in all known nematoceran mitochondrial genomes was found in *Phlebotomus*. However, the typical nematoceran 7-bp overlapping region between *ND4* and *ND4L* was not observed in the mitochondrial genomes of phlebotomine sand flies, in contrast, these two genes overlapped by one nucleotide.

**Fig. 2:**
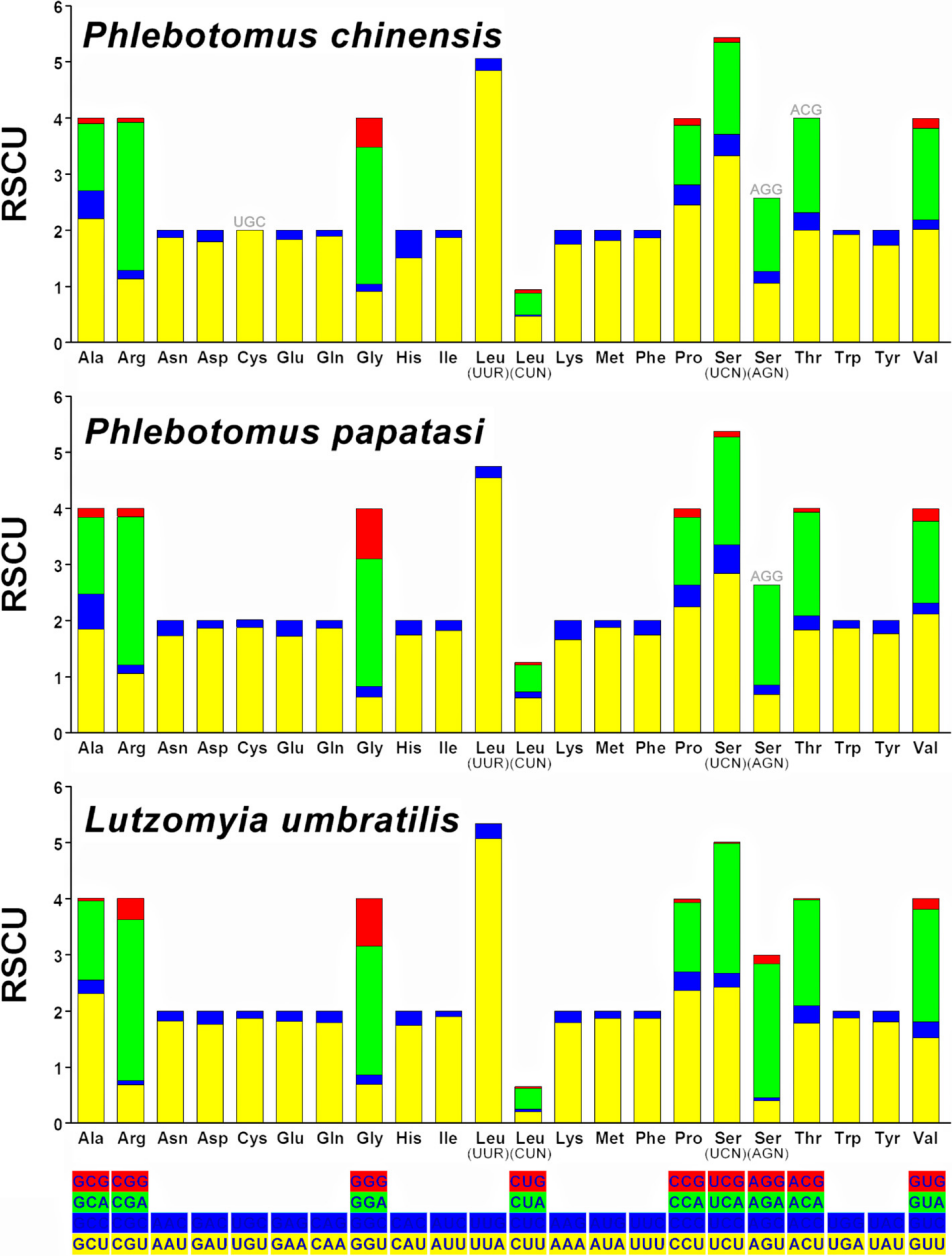
Codon distribution in mitochondrial genomes of *Phlebotomus chinensis*, *Phlebotomus papatasi* and *Lutzomyia umbratilis*. Gray-colored codon indicates codon is not present in the genome

The codon usage patterns of *P. chinensis*, *P. papatasi*, and *L. umbratilis* were summarized and the relative synonymous codon usage (RSCU) values are showed in Fig. 2. In the mitochondrial genome of *P. chinensis*, three codons ACG (Threonine), AGG (Serine), and UGC (Cysteine) are missing, while in the mitochondrial genome of *P. papatasi*, only one codon AGG (Serine) is absent. Overall, all unused codons are rich in G/C. For the mitochondrial genome of *L. umbratilis*, all codons expected codons are present. The significance of an AT-rich genome is reflected in codon usage for mitochondrial proteins. It is clear that codon usage ending with A/T, rather than G/C, is preferred by sand flies. The most frequent amino acids in the PCGs are: Leucine (15.94 %–16.85 %), Isoleucine (10.16 %–10.38 %), Phenylalanine (8.82 %–9.48 %), and Serine (7.39 %–8.80 %). The codons UUA (Leucine), AUU (Isoleucine), UUU (Phenylalanine), and AUA (Methionine) are the most frequently used codons.

### Transfer and Ribosomal RNAs

All typical tRNA genes of metazoan mitochondrial genomes were identified in both *Phlebotomus* mitochondrial genomes studied. All the 22 tRNAs of *P. chinensis*, *P. papatasi*, and *L. umbratilis* have the common cloverleaf secondary structure, while the DHU arm of *trnS*^AGN^ is short with only one complementary base pair. All anticodon usage is identical with that described for other nematoceran mitochondrial genomes, except for *trnS*^AGN^ of *L. umbratilis*, which uses TCT instead of the common GCT. Considering the codon usage, the RSCU of codon AGA (the corresponding codon to anticodon of *trnS*^AGN^) is overwhelmingly higher than those of other three synonymous codons in *L. umbratilis*. The frequency of AGA is moderate rich in *P. chinensis* and *P. papatasi*, however the corresponding codon (AGC) to anticodon (GCT) of their *trnS*^AGN^ is rarely used. The most conserved tRNAs among *P. chinensis*, *P. papatasi*, and *L. umbratilis* are *trnL*^UUR^, *trnL*^CUN^, *trnS*^UCN^ and *trnI*, however *trnA*, *trnR* and *trnC* exhibit low level of identical nucleotides.

**Fig. 3:**
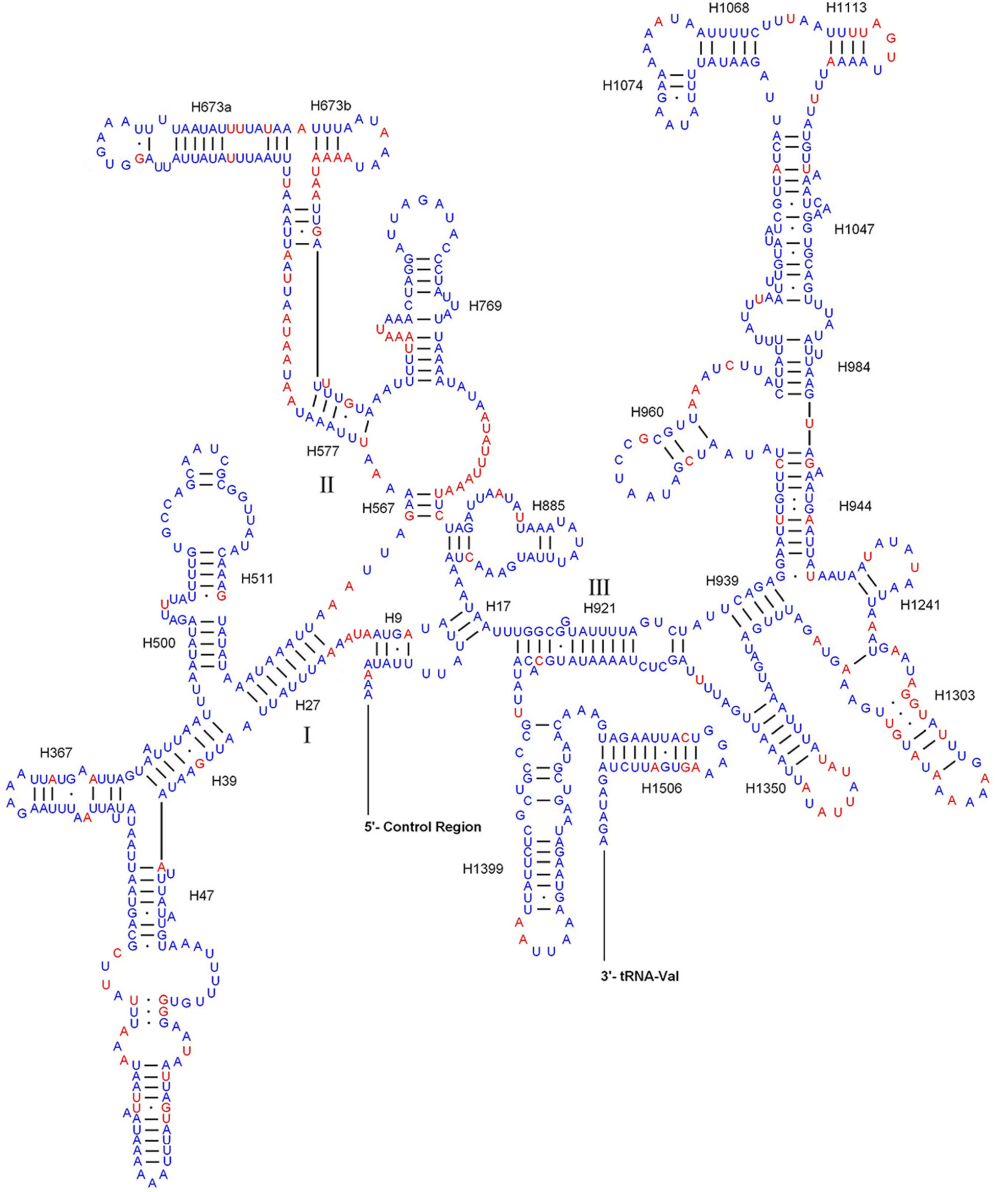
Inferred secondary structure of the mitochondrial *rrnS* gene for *Phlebotomus chinensis* (Conserved nucleotides of three Phlebotominae taxa are labelled in blue)

**Fig. 4:**
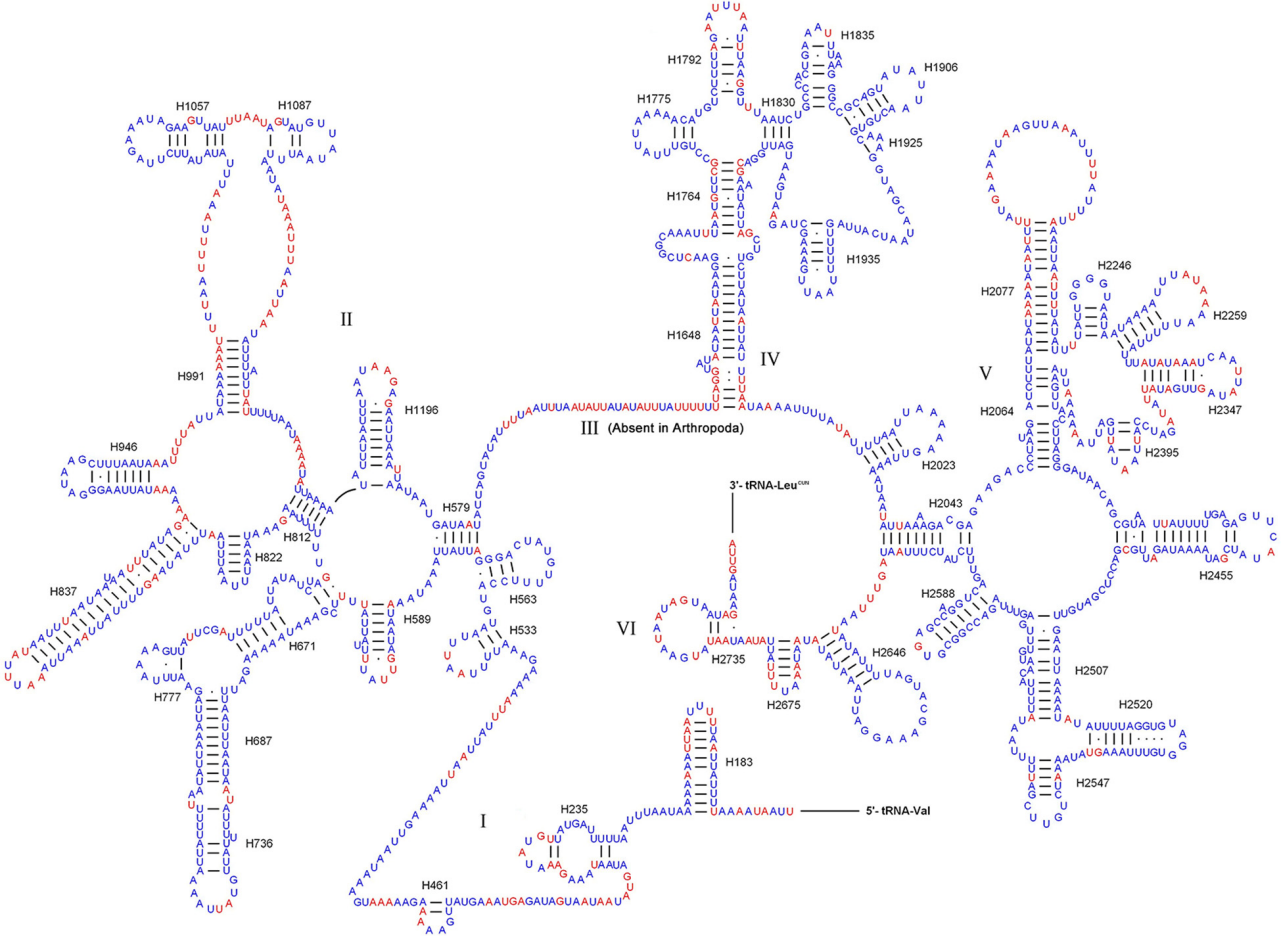
Inferred secondary structure of the mitochondrial *rrnL* gene for *Phlebotomus chinensis* (Conserved nucleotides of three Phlebotominae taxa are labelled in blue)

The inferred secondary structure models of small ribosomal subunit (*rrnS*) and large ribosomal subunit (*rrnL*) for *P. chinensis* are shown in Figs. 3 and 4, respectively. The secondary structure of *rrnS* and *rrnL* contain three and six domains, respectively. The domain III of *rrnL* is absent, which was reported in the secondary structure of other arthropodan *rrnL* [26, 37]. The overall structures of *P. chinensis* rRNAs resemble that of other insects. Comparative analyses on secondary structures among *P. chinensis*, *P. papatasi*, and *L. umbratilis* manifest uneven distribution of conserved nucleotides, in that domains I and III of the *rrnS* are more conserved than domain II, and domains I, II, and VI in *rrnL* have more variable sites. Variable positions of *rrnS* are largely restricted to H47, H673, H1305 and the region between H577 and H673, and H567 and H769. Domains IV and V of *rrnL* contain mainly conserved helixes.

### The A + T-rich region

The A + T-rich regions of *P. chinensis* and *P. papatasi* are 1,433 bp and 723 bp respectively, which harbor a high rate of A + T base composition (91.1 % for *P. chinensis* and 92.3 % for *P. papatasi*). The A + T-rich regions of *P. chinensis* contains seven identical tandem repeat units of 159-bp sequence and another shortened tandem repeat unit with only 79-bp. In *P. papatasi*, there are three tandem repeat units, the first two (162-bp) are nearly identical with one substitution at the 159th position, while the third one is a shortened repeat unit (89-bp). All the tandem repeat sequences of *P. chinensis* and *P. papatasi* begin in the *rrnS* gene, but the tandem repeat sequences (372-bp for repeat unit) of *L. umbratilis* are located in the central region of A + T-rich region. Additionally, the alignments of tandem repeat units of *P. chinensis* and *P. papatasi* show 60.2 % similarity, but there is no evidence for homologous repeat motifs between species of *Phlebotomus* and *L. umbratilis*. Abundant microsatellite-like elements occur throughout the region between the tandem repeat sequence and *trnI* (e.g. (AT)3, (AT)5, (AT)6, (AT)8, (TA)4, and (TA)6 in *P. papatasi*). These tandem repeat units and microsatellite-like elements are potentially useful markers for the study of geographical population structure [38].

The accurate estimation of length and number of repeats and assembly of A + T-rich region are often difficult, particularly for including various complex repeat regions. For obtaining the accurate A + T-rich region of *P. chinensis*, Sanger sequencing with paired ends can cover the length of repeat region (approximate 1.2 kb), and agarose gel electrophoresis for amplified control region was used to determine the correct size and number of the length of repeat region. In control region of *P. papatasi*, we reconstructed the similar pattern of architecture for *P. chinensis*. The high coverage and comparatively long read length also make sequence accurate.

### Nucleotide diversity of mitochondrial genome among *Phlebotomus chinensis*, *P. papatasi* and *Lutzomyia umbratilis*

**Fig. 5:**
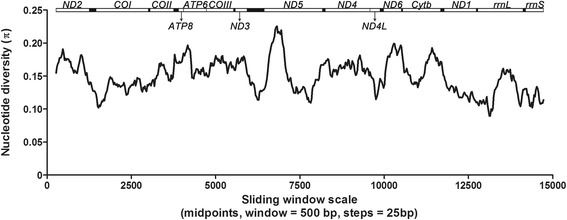
Sliding window analyses of the alignment among *Phlebotomus chinensis*, *Phlebotomus papatasi* and *Lutzomyia umbratilis* mitochondrial genomes. The line shows the value of nucleotide diversity (π) in a sliding window analysis of window size 500 bp with step size 25 bp, the value is inserted at its mid-point

A sliding window analysis was performed to estimate nucleotide diversity Pi (π) across the mitochondrial genomes of *P. chinensis*, *P. papatasi* and *L. umbratilis*, excluding the A + T-rich region (Fig. 5). The sliding window indicated that the most variable coding regions were within *ND5* gene suggesting that these regions are under accelerated evolution and few selective constraints, and can be used as effective markers to investigate population structure and potentially resolve the phylogenetic relationship of closely related species. Not unexpectedly, the overall sequence variability of the rRNA regions is lower than that of other regions. The most conserved fragments were found in the *rrnL* region. Amongst PCGs, *COI* and *ND1* were the most conserved. By contrast, *ND6*, *ATP8* and *ND3* displayed the high variability.

### Phylogenetic analyses

**Fig. 6:**
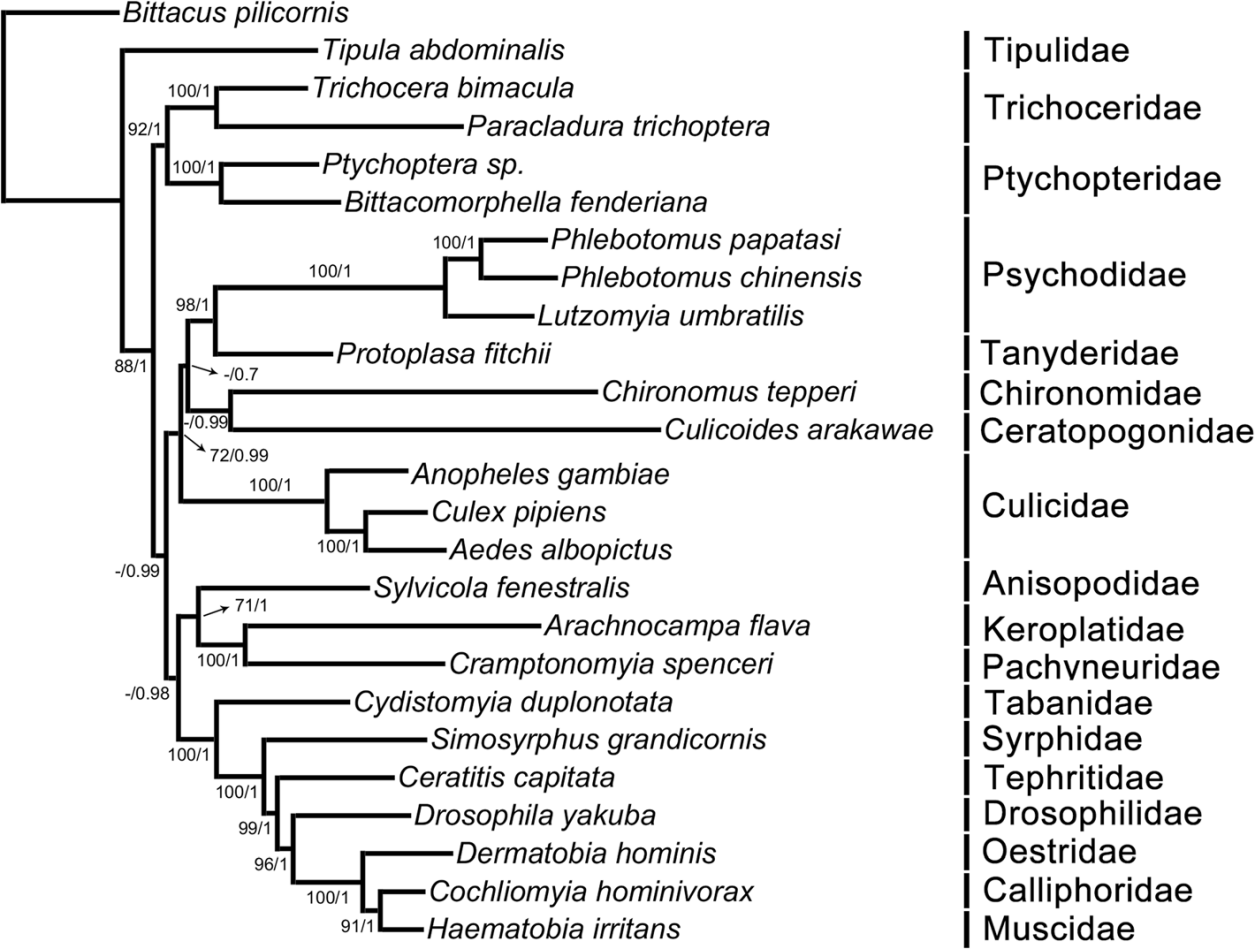
Mitochondrial phylogenetic relationship of representative members of Diptera. First values at the branches correspond to ML bootstrap support in percentages while the second values indicate Bayesian posterior probabilities (ML bootstrap values < 50 % are not shown)

Diptera is a megadiverse group of extant insects. Historically, Diptera was divided into two suborders, Nematocera and Brachycera. Brachycera was confirmed as a monophyletic group with robust phylogenetic analyses, but Nematocera is generally accepted as a paraphyletic group and Brachycera is derived from part of these lineages. The mitochondrial genome contains much information and has been used to resolve the phylogenetic relationships of Diptera, especially that of Brachycera [39–42]. In the present study, the phylogenetic relationships inferred from ML analyses and BI analyses using only first and second codon positions of 13 PCGs share similar topologies (Fig. 6). Consistent with previous results, Brachycera formed a monophyletic group and clustered with Bibionomorpha as the sister group [43, 44]. Surprisingly, Psychodidae species clustered with *Protoplasa fitchii*, the lone representative of Tanyderidae with high support, which is the first time this relationship has been elucidated by mitochondrial data (bootstrap value of 98 % in ML analyses and Bayesian posterior probabilities (Bpp) of 1 in BI analyses) and identical to results of other molecular datasets [43, 44]. This clade was derived from Culicomorpha but the node was weakly supported (<50 % for bootstrap value and 0.7 for Bpp) suggesting the relationship between this branch and Culicomorpha is still ambiguous. However, the close relationship within this large clade was confirmed by moderate node support (72 % for bootstrap value and 0.99 for Bpp), which is in accordance with previous studies using multiple markers [43]. The traditional basal branch comprised of Tipulidae and Trichoceridae (Tipulomorpha) was not grouped as a monophyletic clade, instead Tipulidae was an early split in the phylogeny of Diptera. While the families Ptychopteridae and Trichoceridae formed a branch that clustered with all remaining groups as the sister group. This arrangement of basal branches is identical with 13PCG12 (third codon sites removed) + rRNAs dataset, however 5PCG12 (*COI*-*III*, *Cytb*, and *ATP6*) + rRNAs dataset shows a different topology [34]. However, using different phylogenetic hypotheses caused the topology to change, with Tipulomorpha containing Tipulidae or Tipulidae + Trichoceridae [44–46], therefore we can conclude that the basal placement of Tipulomorpha in the phylogeny of Diptera is stable. Phylogenetic analyses in this study were based only on mitochondrial data, so we believe it is still indispensable to combine nuclear and mitochondrial data with a broader taxon sample to provide an even more robust phylogenetic analyses depicting the evolution of the Diptera.

### Implications

Low flight capacity, a preference to remain close to area of emergence, geographic barriers and variability in climate across their distribution has led to genetically structured populations of phlebotomine sand flies, with cryptic species also being recorded [47, 48]. Genetically distinct species and populations have demonstrated a varying ability to both transmit *Leishmania* and resist insecticides [49–51] highlighting the need to quantify their population structure and delineate cryptic species. The sliding window analysis presented in this study provides a useful comparison of the evolutionary rates of each gene, allowing future researchers to design population genetic and large-scale phylogenetic studies utilizing the most appropriate marker for their task. One immediate use for such data will be the exploration of the relationships between *P. chinensis* and another disputed and close relative vector species *Phlebotomus sichuanensis* or ‘large type of *P. chinensis*’ [52–54]. It is debated whether these two nominal species are in fact distinct or if they are different populations of the same species occupying different altitudes [16, 53, 55].

NGS technology has been routinely used in genomic research with Illumina and 454 platforms. Although, these sequencing technology have been verified to obtain mitochondrial genomes for insects, the A + T-rich region is still difficult to assemble owing to various complex repeat regions [56, 57]. Ramakodi *et al.* [56] reported that the coverage may not have the crucial factors for reconstruction of control region using 454 reads, and known repeat sequences can help to reconstruct the full length of control region. In the present study, we successfully retrieved the complete mitochondrial genome with entire A + T rich region using *P. chinensis* as the reference. Both these control regions contain a similar pattern of repeat sequences, and the repeat units also hold 60.2 % similarity suggesting control region (or repeat sequences) of closely related species may contributes to the reconstruction of a new control region. Furthermore, the results also indicate that mitochondrial genome of closely related species as reference are more appropriate than shot target sequences for reconstruction of the full length of control region, in particular to that including complex repeat sequences. In other words, it suggests the reference species and sequence must be carefully selected when using the same approach. These first *Phlebotomus* mitochondrial genomes will make it easier to generate additional mitochondrial genomes data including control region from different population and species which will provide insight into the speciation, distribution pattern, evolution and divergence times of sand flies at the genome-level [58–61].

## Conclusion

The present study determined the mitochondrial genomes of *P. chinensis* and *P. papatasi*, and conducted a comparative analysis of three sand fly mitochondrial genomes. We present the first examination of the phylogenetic status of the Psychodidae and, based on all mitochondrial PCGs, provide stable support that families Psychodidae and Tanyderidae are sister taxa. We confirmed the known sequences in control region of closely related species facilitate the reconstruction of uncharted control region using the similar approach. Our results also provide a source of genetic markers for future studies on the population biology and molecular phylogeny of these important vectors.
